# Sophisticated Statistics Cannot Compensate for Method Effects If Quantifiable Structure Is Compromised

**DOI:** 10.3389/fpsyg.2022.812963

**Published:** 2022-02-16

**Authors:** Damian P. Birney, Jens F. Beckmann, Nadin Beckmann, Steven E. Stemler

**Affiliations:** ^1^School of Psychology, The University of Sydney, Sydney, NSW, Australia; ^2^School of Education, Durham University, Durham, United Kingdom; ^3^Department of Psychology, Wesleyan University, Middletown, CT, United States

**Keywords:** method effects, reactivity, Rasch measurement, dynamic personality, monotonicity

## Abstract

Researchers rely on psychometric principles when trying to gain understanding of unobservable psychological phenomena disconfounded from the methods used. Psychometric models provide us with tools to support this endeavour, but they are agnostic to the meaning researchers intend to attribute to the data. We define method effects as resulting from actions which weaken the psychometric structure of measurement, and argue that solution to this confounding will ultimately rest on testing whether data collected fit a psychometric model based on a substantive theory, rather than a search for a model that best fits the data. We highlight the importance of taking the notions of fundamental measurement seriously by reviewing distinctions between the Rasch measurement model and more generalised 2PL and 3PL IRT models. We then present two lines of research that highlight considerations of making method effects explicit in experimental designs. First, we contrast the use of experimental manipulations to study measurement reactivity during the assessment of metacognitive processes with factor-analytic research of the same. The former suggests differential performance-facilitating and -inhibiting reactivity as a function of other individual differences, whereas factor-analytic research suggests a ubiquitous monotonically predictive confidence factor. Second, we evaluate differential effects of context and source on within-individual variability indices of personality derived from multiple observations, highlighting again the importance of a structured and theoretically grounded observational framework. We conclude by arguing that substantive variables can act as method effects and should be considered at the time of design rather than after the fact, and without compromising measurement ideals.

## Introduction

We have observed that there is a belief among some that highly sophisticated statistical techniques will be able to correct for fundamental problems in the interpretability of assessment data. Unfortunately, even the most advanced statistical methods remain inert in the face of conceptual negligence. We concur with Michell (1997) who argued that understanding the quantitative structure of attributes is premised on two intertwined research activities: (a) the scientific one, which involves the development of theory regarding the quantifiable structure of the attribute; and (b) the instrumental one, which involves the development of an operationalisation that informs the measurement of the attribute. While there will be iterations between these core activities, the primary task is always the scientific one.

It is generally well-accepted that psychometric measures are prone to influences brought about by the particular method one uses to assess latent psychological attributes. There is an underbelly of belief that extraction of latent variables from multiple measures that use different methods via factor analysis will, in and of itself, purify our assessments from their method effects (however, defined), as well as usefully partial out unintended sources of variability that contribute to unreliability. However, this places an untenable burden on Michell’s instrumental task, in that it obscures rather than illuminates the ultimate scientific endeavour, which is to understand the attribute decoupled from the method used to assess it. This is not some new epiphany. Consistent with van der Maas et al. (2017, p. 3) and others (e.g., Borsboom et al., 2003, 2004), we argue that while the latent variable approach “is straightforward and practical” and “is a very useful data reduction technique” to mitigate complexity in data, it is not well-suited to addressing the validity question at the level of the attribute (Lohman and Ippel, 1993). The validity question is first and foremost a conceptual one. Other “validity concepts,” such as “validity of use” or “validity of prediction” are a distant second, and probably more suitably labelled as predictive utility. Substantive consideration of method effects form part of the scientific task, because it forces an explication of a theory about how the reactivity to a method is manifest in the assessment. Our own approach to managing such effects has been to structure the observational context at the item, person, and situation level (Beckmann, 2010; Birney et al., 2016, 2017; Beckmann and Goode, 2017), and then to model these factors explicitly in the derivation of performance indices.

### Overview

Our overall objective here is to argue that control of method effects will ultimately rest on testing whether data collected fit the model or theory proposed (based on a substantive theory about the attribute), rather than a search for a statistical model that best fits the data after the fact. In short, we advocate data fit rather than model fit, which needs to be embraced at the point of design and stated explicitly in reporting empirical research. Paraphrasing Pedhazuer and Schmelkin (1991, p. 2), no amount of psychometric sophistication will absolve us of the responsibility of thinking first.

We aim to demonstrate (1) that psychometric sophistication does not necessarily equate to better measurement, and (2) by drawing on two applied case study examples, that method effects are pervasive, and accordingly should be factored into consideration as part of the structural design of the measures, *and* as part of the study design. We begin this review by first reminding ourselves of the importance of measurement models (Kellen et al., 2021; Stemler and Naples, 2021) and highlighting the distinction between the Rasch measurement model and the general IRT approach, both of which of course are inherently latent variable measurement models. We do this in an attempt to demonstrate the slippery psychometric slope one can find oneself on when the balance between data and theory is misaligned or too heavily informed by pragmatics, such as attempting to control for the potential impact of extraneous factors. Central to this first section is the consideration of the concept of fundamental measurement as defined by Luce and Tukey (1964) and elaborated on by Michell (1990) and others in terms of additivity of measurement (e.g., Brogden, 1977; Perline et al., 1979; Wright, 1999). Psychometric corrections for method effects should not compromise the validity of the structure of measurement. This may seem an odd (or counter-intuitive) statement to make, given the primary purpose of such corrections is often “to improve” validity. However, this intention is not always achieved (van der Maas et al., 2017; Broers, 2021; Kellen et al., 2021). For the purpose of this article, we propose that what qualifies as a method effect can be conceptualised as follows:

If an action* external to a measure, whether by design or otherwise, results in a weakening of the quantitative structure of that measure through (a) a change in the psychometric properties of the attribute assessed, or (b) a change in the actual attribute assessed, then it is a method effect.

* Importantly, this “action” could be a theoretically substantive and intended manipulation, a statistical modelling decision, or some other facet of the assessment circumstances, whether it be an intended one or not.

We then report on two lines of research as our case studies of substantive method factors, which we argue impact validity of measurement. First, we summarise investigations of the reactivity to metacognitive probes and their effects on the assessment of cognitive performance. As it turns out, leading someone to reflect on their performance on a previously attempted item at a metacognitive level can impact their subsequent performance (Fox et al., 2011; Birney et al., 2017; Double and Birney, 2019a). This experimental approach to method effects is then compared with the complementary factor-analytic investigation of metacognition (Stankov, 1998). Second, we reflect on research which considers outcomes from multiple momentary assessments of personality. These repeated observations are not taken simply to improve reliability of measurement, although this is obviously important. Rather, our observational design is structured so as to systematically assess latent attributes across different occasions, situations, and sources in terms of the observed level of the attribute (e.g., sum-scores), but also its variability (Birney et al., 2017; Beckmann et al., 2020, 2021).

## Measurement Models and Method Effects

The merits of fitting data to models, versus fitting models to data is contentious. What we summarise here in this section is far from new, but it does remind us of the importance of theory, even when it comes to considering the treatment of method effects. To begin, we disclose our position… we propound the merits of fitting data to models and acknowledge the temptation of fitting models to data, but also recognise that whereas model fitting may have an informative role early in theory development, there are good reasons why it should not be the basis of measurement. The reason for this is based in part on the importance of measures approximating additivity – the ideal of which is fundamental measurement. We now take a brief diversion to consider this.

### Fundamental Measurement

Our use of the term “fundamental measurement” is in relation to the additivity of the quantitative structure of an assessed attribute. Luce and Tukey (1964) outline the principles of additive conjoint measurement as a means of assessing additivity. Michell (1990) summarises the argument for conjoint measurement in psychology and the logical “cancellation” conditions that need to be met in order to support claims of additivity. In short, conjoint measurement is concerned with the way the ordering of a dependent variable varies with the joint effect of two or more independent variables (e.g., task manipulations). To explicate and following Michell (1990), assume that *R* represents the *ordering of differences* in reasoning demands (e.g., task complexity), and that *S* represents, for instance, the *ordering of differences* in short-term memory demands. The observed dependent variable, *P* represents performance on an appropriate measure along which the effect of *R* and *S* is assessed. Accordingly, the *ordering of R and S* is necessarily dependent upon the *order of P*. That is, their orders are relative to their effect on *P*, and therefore, the independent variables *R* and *S* are quantified relative to their effects on *P* (Perline et al., 1979; Michell, 1990). The orderings across specific levels of R and S need to follow specific monotonic relations, referred to as “cancellation” criteria, to satisfy principles of additivity. Scheiblechner (1999) provides a detailed account of how cancellation principles can be applied to link subject and item-parameters (see also, Scheiblechner, 1995). While it is beyond our scope to go into these details, a relevant application is reported in Stankov and Cregan (1993). They used conjoint measurement principles to assess the quantitative characteristics of fluid intelligence in relation to motivation and working memory demand (i.e., task complexity). From their results, Stankov and Cregan (1993) concluded that intelligence has quantitative structure.

Conjoint measurement offers a deterministic account (Brogden, 1977). If the necessary cancellation conditions are met then quantitative structure is supported, otherwise no valid conclusion can be made about the nature of the scale. Perline et al. (1979) point out how Rasch analysis overcomes to some extent the deterministic nature of conjoint measurement. By allowing for a stochastic assessment of quantity that can be tested for goodness of fit, a probabilistic estimate of the likelihood that conjoint measurement exists can be obtained when not all cancellation conditions are met. The IRT approach generally attempts to jointly map both the individuals’ ability and item difficulty on the same underlying metric. A satisfactory fit of the data to the Rasch model is reported to demonstrate additivity of measurement (Brogden, 1977). Wright (1999) argues that this implies that an interval scale of measurement has been achieved.

In the next section, we make a comparison between the Rasch and IRT models to explicate a distinction between measurement attempts that follow a principled approach to fundamental measurement and those which follow more pragmatic model-search-and-fit approaches. We note that for this purpose, the Rasch measurement model, as well as other parametric (e.g., 1PL-IRT) or non-parametric IRT model (Junker and Sijtsma, 2000) would suffice as long as the models implemented are proposed in advance (i.e., theory before data) and importantly, restrict item response functions to be ordered monotonically.

### Differential Item Discrimination as a Method Effect

**FIGURE 1:**
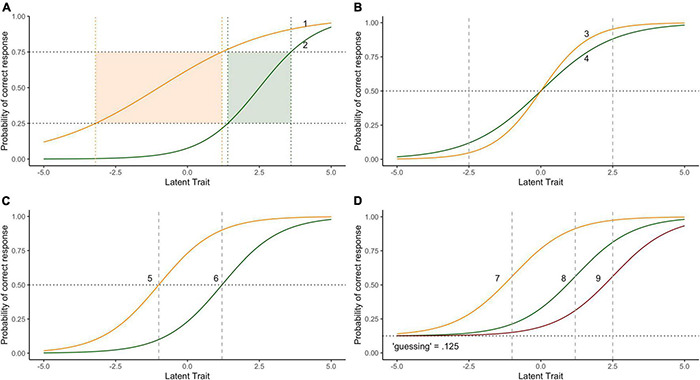
Item response functions reflecting different models **(A)** differential item discrimination with non-overlapping curves, **(B)** differential item discrimination with overlapping curves, **(C)** constant item differentiation, **(D)** constant differentiation and adjustment for “guessing.”

When it comes to test development and measurement using IRT methods, the data fit vs. model fit decision often equates to “Rasch or IRT.” It arises when one considers, for instance, whether to allow item-discrimination slopes to not be uniform and potentially overlap (as in 2PL IRT models), or to constrain them to be equal and not overlap, as in the Rasch Model^1^. A further issue is whether to allow the lower asymptote to be greater than 0 (allowing for “pseudo-guessing,” as in 3PL IRT models). Psychometrically and pragmatically, the notion of differential item discriminability is appealing. It makes sense that some items might be more or less precise in differentiating individuals across the ability continuum than others. For instance, the item characteristic curves represented in Figure 1A demonstrate that for Item 1 (which has a discrimination slope of 0.5), there is a change of 4 logits across the latent trait scale (from −3.2 to 1.2) to move from *p* = 0.25 to *p* = 0.75 probability of a correct response.

In contrast, across the same probability range Item 2 (with a discrimination slope of 1.0) spans only a 2.2 logit change in the latent trait. What this suggests is that Item 2 is a much more effective and precise contributor to differentiating ability than Item 1. In practice, a good test would have more items similar to Item 2 than Item 1 spread out to span the latent-trait continuum in order to derive a precise differentiation of ability levels. Without a clear process theory of how latent attributes impact responses at the individual item level, this, however, can be challenging. An alternative approach is to keep the “good, but not great” items we have and find a model with a set of parameters that gives an appropriate weight to better discriminating items. However, this “psychometric flexibility” comes at a cost to the adherence to the principles of fundamental measurement.

The item functions in Figure 1A obscure the main problem of allowing item discrimination to vary because in this case they do not overlap, whereas Items 3 and 4 in Figure 1B for instance do. Briefly, we can see that for the cohort of test-takers with latent-trait ability <0, the probability of a correct response is greater for Item 4 than Item 3 (Item 3 is more difficult than Item 4). On the other hand, for the cohort of test-takers of higher ability (with latent-trait >0), Item 3 is easier than Item 4. That the relative probability of a correct response to one item relative to another depends on ones’ ability in this way is inconsistent with the notions of fundamental measurement (Masters, 1988; Scheiblechner, 1995; Andrich, 2003; Stemler and Naples, 2021). The measurement assumption, simply stated, is that if Person A has more of the underlying latent trait than Person B, then the probability of a correct response will *always* favour Person A. If there are circumstances where this is not the case, then something is going awry, and whatever that something else might be, it threatens the validity of the items (and therefore test) to assess the quantitative structure of the latent trait. That we have psychometric models that allow for different slopes for each item in our test is impressive, but the theoretical implication is that we need to admit that we do not well-know in advance how the task features interact with the to-be-measured attribute to influence a response. The only solution to this threat to validity of measurement is to require item function slopes to be uniform and monotonic, such as Items 5 and 6 (Figure 1C), and to construct items consistent with this^2^. The Rasch model is an example of such a monotonic model, although there are others, including non-parametric ones (e.g., Scheiblechner, 1999; Junker and Sijtsma, 2000).

Finally, for completeness, a third parameter often considered in IRT models aims to take into consideration the propensity for someone of asymptotically low ability to still respond correctly to an item. This concept is well understood for multiple-choice type items, where there is greater than zero probability of an individual selecting the correct option by chance, but can also apply differently to different items in the test beyond the number of options available to choose (e.g., the wording of some items might increase the chances of a correct response independent of ability) [though see Wright (1988), for alternative views on this]. Items 7–9 (Figure 1D) represent the case where there is a constant slope (discrimination), a constant guessing parameter (eight options: chance *p* = 0.125) and variable item-difficulty^3^. Where we have structured measurement observations such as we have here, it is appropriate to recognise this structure in advance with an appropriate measurement model, such as the one presented. In practice, due to the specific-objectivity of the Rasch model permitted by the lower-asymptotes being constant, the item- and person-calibrations are monotonically ordered within a linear transformation (e.g., Embretson and Reise, 2000).

Stemler and Naples (2021) demonstrate that the choice of whether to allow items to vary in discrimination is not simply a matter of psychometric preference. Their simulations show that ability estimates from the same people based on models where item-discriminations are allowed to vary (common 2+PL IRT models) can differ substantially from Rasch ability estimates, where slopes (and guessing) are constant. Whereas IRT estimates are by definition heavily sample and item dependent, Rasch estimates are not, at least not when there is acceptable fit of the data to the model (Wright, 1977). While general IRT approaches are designed to better fit data, the cost is significant both theoretically from a fundamental measurement perspective, but also, Stemler and Naples (2021) argue, because it has implications for practical use and interpretation, for instance in criterion-based assessments.

#### Comparison With Classical Test Theory Approaches

Whether one allows items to differ in discrimination is not a focal issue in CTT. This is not because of any special feature of CTT, but rather because the underlying measurement model, typically a congeneric one, focuses on optimising psychometrics properties of the item composite (e.g., sum-score) in a rather data-driven manner. Accordingly, in CTT, items are not fundamental but selected for their statistical value (Embretson and Reise, 2000) without an inherent test of their quantitative data structure (Broers, 2021). This is despite the fact items are central to notions of reliability of measurement in CTT models. To demonstrate this, consider the differences between the familiar Parallel, Tau-Equivalent, Essentially-Tau Equivalent, and Congeneric measurement models. While all models assume unidimensionality (whether it is tested or not), each places different requirements on the item data which makes up the composite test score. Graham (2006) represents these different models, ordered from most to least restrictive, as follows (where X*_*ik*_* is the observed score for person *i* on item *k*, *T* = True score and *E* = error):


$$\text{Parallel:}X_{ik}=T_{i}+E_{i}$$

$$\text{Tau-Equivalent:}X_{ik}=T_{i}+E_{ik}$$

$$\text{Essentially Tau-Equivalent:}X_{ik}=\left(\alpha_{K}+T_{i}\right)+E_{ik}$$

$$\text{Congeneric:}X_{ik}=\left[\alpha_{k}+\beta_{k}\left(T_{i}\right)\right]+E_{ik}$$

As summarised by Graham (2006) and others (e.g., Pedhazuer and Schmelkin, 1991), it can be noted that parallel measurement models are the most restrictive, *inter alia* requiring all items to be unidimensional (i.e., a single latent variable), measured on the same scale (i.e., equal variance), with equal item-specific errors, and to the same degree of precision (i.e., equal item True scores). Tau-equivalent and Essentially Tau-Equivalent models allow item-specific errors, and both item-specific errors and item-precision to differ [i.e., by the constant α_K,_ see Graham (2006) for further details], respectively. Parallel and Tau-Equivalent models are roughly analogous to the Rasch measurement model in that the k item true scores (T*_*i*_*) are equal (or differ by an additive constant, as in the Essentially Tau Equivalent model). The congeneric model on the other hand, allows for the scale of the *k* item true scores to differ by a factor of β*_*k*_*, as well as allowing difference in item-specific error and precision. Items are therefore not constrained to be monotonically ordered, in much the same way items in 2PL-IRT models are not. Item parameters and their inter-relations are free to vary from sample to sample as the data dictates. This psychometric freedom bring into question the consistency of measure across occasions, and thus also test-validity (Kellen et al., 2021).

To sum up our position so far, the derivation of ability estimates from items such as 7–9 (Figure 1D) are to be preferred for two reasons. First, they equate to a model where the lower-bound has been adjusted because of the multiple-choice method chosen, while at the same time prioritising specific objectivity of measurement. While we have taken some space to explicate our position in favour of the Rasch model, the point is this: Method effects should be planned for and built into the structure of the ability estimation procedure *in advance* of collecting the data, rather than being determined in sample- and test-dependent ways *post hoc*.

**FIGURE 2:**
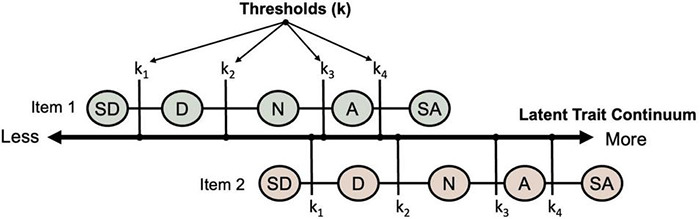
Rating scale thresholds (where SD = strongly disagree; D = disagree; N = neutral; A = agree, and SA = strongly agree; each is assigned a numerical ordering of 1–5, respectively). The response category thresholds are represented along the latent continuum of the attribute.

Before moving on, it is illustrative to address how the Rasch measurement model deals with polytomous item responses while maintaining additivity/monotonicity. The classic example is the self-report rating scale (Andrich, 2016; Bond et al., 2020), where, for instance, verbal descriptors of level of agreement to item statements are assigned numerical values (e.g., strongly disagree = 1 through to strongly agree = 5). Similar to the known potentiality of guessing in multiple-choice items, rating scale items that span a latent trait continuum (such as in Figure 2) introduce a number of pragmatic challenges to measurement. Solutions to some of these challenges have been established for some time (e.g., Andrich, 1978; Masters, 1982). For instance, the allocation of a given rating to one question (e.g., *neutral* to Item 1) may reflect the same amount of latent attribute as a different rating to another question (e.g., *strongly disagree* to Item 2); and the relative difference in the attribute needed for, say, a *strongly disagree* (1) versus a *disagree* (2) response may not reflect the difference between *agree* (4) and *strongly agree* (5), although the differences of the numerical values assigned to them are equal. That is, at the item-response level, there is no additivity of differences, a tenet of fundamental measurement (Luce and Tukey, 1964; Michell, 1990). Andrich’s rating scale approach models the additional amount of the attribute needed to move between response categories (thresholds, k) to differ (i.e., k_2_ – k_1_ *can be* different to k_4_ – k_3_), but holds these relative threshold differences constant across all items in the test (i.e., as in Figure 2, the difference between k_1_ and k_2_ is the same for Item 1 as it is for Item 2) and across all respondents. Now, this flexibility may be seen as opening us up to similar model-fitting criticism we have raised against the general IRT approach (as compared with the Rasch approach). But the comparison is not equivalent, because additivity of the item components – difficulty and response category thresholds – and therefore specific objectivity, is maintained (i.e., item discrimination curves will not overlap).

Given this position on aspiring to fundamental measurement, we now move to our first applied case study of method effects in terms of assessing the nature of metacognition in cognitive tasks.

## Reactivity as a Method Effect

One of the core method effects of concern for psychology is the potential for test-takers to react in some way to the fact that they are being measured (Double and Birney, 2019b). This effect has been referred to as “reactivity.” Various well-worn test-design practices have long been used to mitigate what might be considered common causes of reactivity, that through aggregation, help to wash-out measurement “noise” to varying extents (e.g., structured item-writing principles, using multiple items and counter-balancing, and employing standardised test administration). There are two ways that reactivity can be observed. The first is as a differential reaction reflected in an item × person interaction across the course of the test. This sort of reactivity has been investigated as experience effects, for instances as learning trajectories using MLM approaches (Birney et al., 2017), or item-position effects using fixed-links SEM approaches (Schweizer, 2006; Ren et al., 2012). At the heart of this trajectory-focused work is a higher-level challenge to the assumed unidimensionality of the item-sum-score. It forces investigation of the possibility of substantive “experience effects” that can be hidden through the item aggregation process (Bui and Birney, 2014). A “get-out-of-jail-free card” is to embrace the typical assumption that experience-effects are determined by the same latent attribute targeted by each of the individual items. That is, for instance, that the fluid intelligence underlying the induction of Ravens rules from earlier items is the same fluid intelligence that is applied to adapt those prior inductions to solve later items. If this is the case, then there is little risk to the validity of the unidimensionality assumption. If it is not the case, that is, if experience effects are moderated by one or more attributes other than the targeted one, validity is threatened [see Birney and Sternberg (2006, p. 318), for further consideration of this].

Experience effects have also been at the core of the so-called *learning test* approach. In learning tests, after an incorrect response to an item, test takers receive error-specific feedback and thinking prompts (Guthke and Beckmann, 2000). Subsequently, test takers are given the opportunity to apply insights (or learnings) gained from processing said feedback on subsequent items. The diagnostic focus in these tests lies on test takers’ responsiveness to feedback (Guthke and Beckmann, 2003; Beckmann, 2006). In other words, test takers’ response to an (item-by-item) intervention can be conceptualised as a “positive” or desired form of reactivity.

The second way that reactivity can be observed is by imposing an explicit observational structure on to the measurement process (Lohman and Ippel, 1993). This can be realised through experimental manipulation, for instance by using a complexity theory to structure item development (Birney et al., 2012, 2019), or more indirectly during the course of investigating other underlying processes. An example of the latter is reflected in endeavours to evaluate metacognitive processes by using participant think-aloud protocols (Fox and Charness, 2010; Fox et al., 2011) or requesting contemporaneous (aka online) self-reports of confidence ratings after each item response, which we refer to as post-item-prompts (Stankov, 2000; Jackson and Kleitman, 2014; Stankov and Lee, 2015). It is instructive to further unpack this second type of reactivity because it highlights an important difference between the correlational and experimental approaches to validity, and the way each (implicitly or explicitly) deals with method effects.

### Reactivity of Metacognitive Prompts

Working from the post-item-prompt paradigm, Double and Birney (2019b) report that the common explanation for reactivity is that prompts for an evaluation of self-monitoring trigger metacognitive processes (in addition to task-directed cognitive processes) that lead test-takers to attend to internal cognitions in a way they would not have ordinarily done. While there is evidence of both positive (performance enhancing) and negative (performance inhibiting) reactivity under these sorts of circumstances, the more important finding is that their impact tends to be moderated by individual differences (see also, Beckmann et al., 2009). For instance, after controlling for general reasoning ability, Birney et al. (2017) found that when a sample of senior managers were asked to provide confidence ratings after solving each of the 36 Raven’s Advanced Progressive Matrices items, they performed significantly poorer overall – a form of negative reactivity – than those who attempted the items without confidence ratings. Also after controlling for general reasoning ability, in a series of studies, Double and Birney observed that negative reactivity was more likely in those with low pre-existing confidence levels (Double and Birney, 2018), that confidence prompts encourage reasoners to be more performance-, rather than mastery-focused (Double and Birney, 2017b), and that confidence prompts lead to better performance (i.e., positive reactivity) in those already with high levels of pre-existing confidence, but not those of lower pre-existing-confidence, which was impaired (Double and Birney, 2017a). In this work, pre-existing confidence levels were task-specific and self-reported after attempting a small number of practice items of the same type.

To be clear, we do not consider confidence as a form of reactivity, rather we present this line of research as a case-in-point where asking participants to rate their confidence *results* in reactivity in measurement. That is, a change in performance (i.e., behaviour) on a task of which participants are being asked to rate their confidence. The approach we have used to collect confidence ratings follows the seminal work of Stankov (1998, 1999, 2000) and colleagues (e.g., Pallier et al., 2002; Kleitman and Stankov, 2007). In this work, mean item-level confidence ratings from multiple tests are collated across a range of different broad ability factors (e.g., fluid, crystallised, and spatial intelligence) and calibration biases related to differences in relative mean confidence and mean accuracy are compared. When factor-analysed alongside task performance accuracy, the extant research (some of which is cited above) has routinely demonstrated evidence for a common confidence factor (i.e., trait-confidence) related to but distinct from the broad-ability factors. Stankov and Lee (2015) summarise a growing evidence base in favour of this “*g*” *factor of confidence*, and have suggested it has a ubiquitous status similar to the “g” factor of intelligence, and accordingly should be more fully considered.

As far as we are aware, no research has yet been conducted to investigate whether individual differences in such a unidimensional trait-confidence factor can account for the range and type of reactivity reported in the quasi-experimental literature^4^ (e.g., Mitchum et al., 2016; Double and Birney, 2019b). At one level it is hard to reconcile the range of positive and negative reactivity observed in the experimental research with the evidence for a positive manifold reported by Stankov and others (e.g., Jackson and Kleitman, 2014). However, it may be the case that because trait-confidence is operationalised as the common factor derived from an aggregation across various ability tests, it is not sensitive to the less frequent exceptions of negative reactivity observed in experimental work. If scientific research tells us anything, it is that exceptions warrant close investigation. The evidence for both positive and negative reactivity would suggest that criticisms of the cognitive “g” factor might similarly apply to a confidence “g” factor. That is, that the general factor is an interesting epiphenomenon of the measurement process and potentially nothing more than a statistical artefact with diminishing substantive value [see Sternberg and Grigorenko (2002), for different perspectives on the meaning of the cognitive “g” factor]. Further research is necessary to determine the boundaries of comparison between experimental and correlational research in this area.

Importantly for the current discussion, the conditions under which positive and negative reactivity is observed in task performance are potential triggers for disordered measurement of the latent trait. That is, if we accept the assumption that the latent trait remains unchanged during assessment, and that it is only task performance that is systematically moderated when people are asked to provide confidence ratings (but not otherwise), then monotonicity of person ability estimates will of course be compromised. The extent of the compromise will depend on the person’s position on the moderating variable when confidence ratings are required, but not otherwise (or at least not in the same way).

In the next section, we consider the case for exceptions more closely as it applies to our work on dynamic personality. We report on how we have taken on the challenge of quantifying method effects in what could be also considered a form of reactivity, but one that is due to the form, context and source of assessments. Our approach is to explicate these facets in our experimental design and then directly consider them as planned model parameters. In sum, we will argue that if one is to accept that momentary assessments of psychological states are contingent on the context in which responses are recorded, which we do, then collecting information about the context and incorporating these into the measurement model is critical.

## Dynamic Personality: Within-Person Accounts of Form, Context, Source and Time

The dominant unit of observation in personality assessment are self-reports. The inherent subjectivity of self-report means that the field has had to address its fair share of method effects (faking, for instance, is a significant challenge). Somewhat consistent with notions of reactivity, we have recently reported on a series of studies to try and better understand the dynamic components of psychological attributes, especially personality, in different contexts and circumstance (Beckmann et al., 2010, 2020, 2021; Minbashian et al., 2010; Wood et al., 2019). In Beckmann et al. (2020) we were interested in the assessment of personality attributes of a sample of business managers in either work and non-work situations (*context factors*) as rated by themselves (i.e., self) and as rated by others who knew them either through their work or non-work settings (i.e., *source factors*). It is this latter work that we review here. Researchers of dynamic personality start from the increasingly well accepted position that between-person rank-order stability co-exists with within-person change in responses under different conditions and over time (Minbashian et al., 2010; Geukes et al., 2017; Vazire and Sherman, 2017). In doing so, the notion of variability as an *individual difference* factor needs to be explicated (Fiske and Rice, 1955; Salthouse, 2012; Lievens et al., 2018; Birney et al., 2019), which is what we outline next.

### Variability as an Individual Difference Factor

First, in our work we tend to conceptualise the variability in repeated assessment under different circumstances using mixed-effects multi-level models. In Beckmann et al. (2020), individual item ratings were considered the unit of observation, and modelled as trait-level (mean), and as intra-individual variability across different contexts. Our operationalisation of variability in item response was based on the notion that the consistency with which a participant responds to different items of the same personality scale can be conceived as a measure of an intra-individual variability “trait.” To redress the effect of the boundedness of the trait scale that results in a functional dependency of variability from the mean, we used the Mestdagh et al. (2018) relative variability index. This index as implemented in the associated R package (Mestdagh, 2016), reflects the proportion of observed variability relative to the maximum possible variability given the participant’s observed mean. In doing so, we aimed to acknowledge that different items represent different manifestations of personality under different situations, and thus offers a source for additional information over and above the sum-score trait-level that is typically used.

We asked participants to indicate how accurately different terms (such as “Moody” or “Adventurous”) describe them (or for “others,” how it describes the person they have been asked to rate). These were done in reference to three contexts, in general (i.e., no specific context), at work, and in non-work situations. One could argue that providing a context triggers reflection of past circumstances, or general impressions of situations in which they have experienced being, say, “Moody” or “Adventurous.” Importantly, when it came to the other raters (who were chosen by the participant), each provided evaluations in contexts that they were most familiar with the participant (i.e., in work or non-work settings). The goal of the research was to investigate the utility of these different indicators under different contexts in predicting job-performance, although here we will focus only on the ratings. In total, each of the 288 participants therefore contributed 12 (items) × 5 (dimensions: Openness, Conscientiousness, Extraversion, Agreeableness, and Neuroticism) × 2 (contexts, work/non-work) × 2 (sources, self/other) = 240 observations for analysis.

The procedure we have just described provides a structured basis for observation which makes our expected method effects explicit. At one level our approach is an example of multi-trait-multi-method design, with five different traits (personality dimensions) and three sets of different methods (1) level vs. intra-individual variability, (2) self vs. other sources of observations, and (3) work vs. non-work contexts. In Beckmann et al. (2020), we modelled this structure accordingly. Our first set of analyses explored the degree of systematicity in the personality indices as a function of the contextual frame and source. Details of the analyses can be found in the article, but this systematicity can also be observed by visual inspection.

**FIGURE 3:**
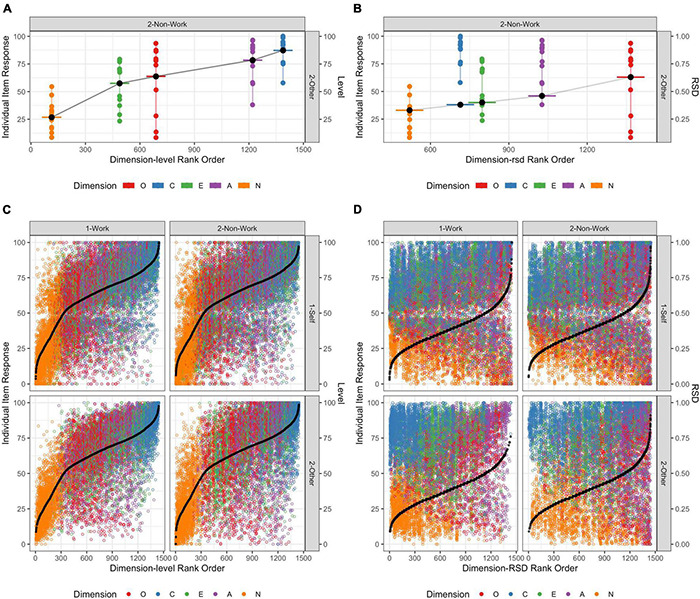
Representation of response variability: Plot of item response by source (self vs. other) and context (work vs. non-work) for each item from five personality dimensions, ordered by **(A,C)** participant mean level, and **(B,D)** participant intra-individual variability (relative standard deviation, RSD). **(A,B)** Are for a randomly selected participant for illustration, whereas **(C,D)** are for all respondents.

Each plotted point in Figure 3 represents a rating on a visual analogue scale of 0–100 provided by a participant (“1-Self”) or their informant (“2-Other”), on one of the 12 items from one of the 5 personality dimensions, in work (“1-Work”) or non-work (“2 = Non-Work”) settings. While in principle 69,120 observations were possible, due to missingness, the 57,516 separate observations available are plotted across the four context (work/non-work) × source (self/other) combinations [see Beckmann et al. (2020) for details].

For illustration, Figures 3A,B represents one randomly chosen participant. In these plots the five personality dimensions are represented by different colours, and each dot represents the actual rating provided for each of the 12 items from that dimension. In Figure 3A, the participant’s mean level across these 12 items for each dimension is represented by the black dot and coloured horizontal bar. The participants responses are ordered along the *x*-axis according to the rank order of their mean dimension level scores relative to other participants’ dimension-level scores for the given context and source [which in (A) and (B) are non-work and other, respectively]. Figure 3B plots the same response data (for the same participant), however, the black dot and coloured horizontal bar now represents the estimated *relative standard deviation* (RSD) according to Mestdagh et al. (2018) which has a scale from 0 to 1 (Mestdagh, 2016), and indicated here on the right-hand *Y*-axis. Figures 3C,D are respectively analogous to Figures 3A,B for data from all participants and with all contexts and sources included.

**FIGURE 4:**
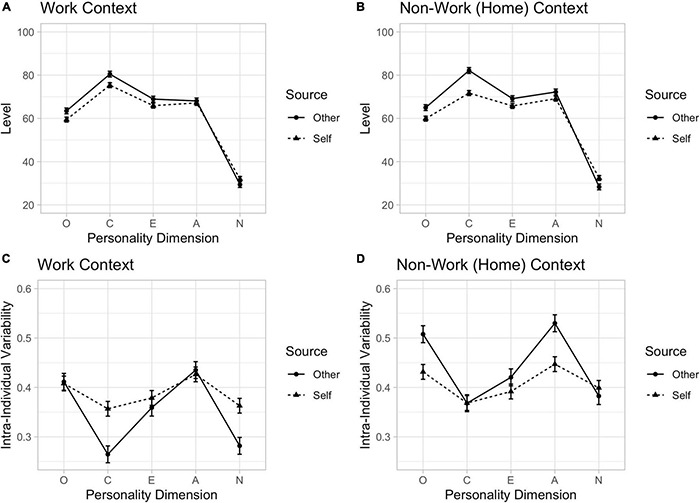
Participant personality dimension mean in work **(A)** and non-work **(B)** contexts and intra-individual variability in work **(C)** and non-work **(D)** contexts, by self and others. Potential variable range: level = 0–100; intra-individual variability (relative standard deviation) = 0–1.

We can see that for traditional measures on which between-person comparisons are typically operationalised (i.e., mean dimension level, Figures 3A,C), there is relatively little differences due to context and source. This can also be observed when the mean dimension level scores are considered, as presented in Figures 4A,B. On the other hand, when intra-individual variability is considered, context and source effects are more apparent in both the distributions of responses (Figures 3B,D) and in the summary dimension intra-individual variability scores (Figures 4C,D).

Details of the analytic approach and theoretical implications are reported in Beckmann et al. (2020), here our point is not on why there are differences, but simply that there are differences. Outcomes of our structural design are summarised in Figure 4. Figures 4A,B plot the overall mean self and other response for each personality dimension in work and non-work contexts, respectively. As can be seen, there are relatively systematic effects. On average, participants tended to rate themselves higher on Neuroticism and lower on the remaining dimensions than others rated them, and this did not depend on whether the context was work (Figure 4A) or non-work (Figure 4B). Thus, while the source of the rating seems to matter, the context did not. In contrast to this, we observed considerably more divergence in intra-individual variability (Figures 4C,D, which is a summary of the data in Figure 3D). First, there tended to be more variability in ratings in non-work settings (Figure 4D) than work settings (Figure 4C). This makes some intuitive sense, given the relative heterogeneity in the types of experiences one has available to reflect on in deriving a response to a given behaviour (e.g., “Adventurous”) in non-work compared to work situations (the latter are rather homogenous in this regard). Second, there was greater divergence in the intra-individual variability in how participant rated themselves and how others rated them, and this differed by context. In work contexts, others tended to report less variability than the self. In non-work settings, the opposite was observed. Others tended to report more variability in their rating of the participant than the participant did themself. What is also worthy of note is that alignments between self and others occurred for different personality dimensions in work settings compared to non-work settings. Aspects of these details are further discussed in Beckmann et al; the point we wish to make here is that the systematic patterns of homogeneity and heterogeneity observed in intra-individual variability as a function of context, source, and personality dimension is largely obscured using more traditional observational designs (aggregating across items). That is, not only is it important to have an appropriate observational design, the choice of the measure operationalised from that design remains a critical part of the scientific task (Michell, 1997).

## Conclusion

Our core objective was to highlight that any solution to managing method effects, whether it be by factor analysis, item response theory, or mixed-effects multi-level models, will ultimately rest on testing whether data collected fits the expected underlying quantitative nature of the attribute. To put this another way, sound measurement theory should inform the operationalisation of the attribute and it should be based on a substantive understanding of its latent structure. Our argument is that while on the one hand “method factors” as systematic sources of individual differences can certainly be a threat to validity and must be controlled for appropriately, that control should be planned prior to data collection rather than crafted to suit the data once collected. Should this not be possible, it then highlights a weakness in the conceptual foundation of our measurement attempts in the particular domain, which cannot be compensated for by more sophisticated statistical procedures.

In describing and explicating the rationale for making a distinction between the Rasch measurement model and more generalised item response theory models, we have sought to make the case for taking the notions of fundamental measurement seriously. The “measurement model” employed should not be a matter of choosing the one from a repertoire of models which best fits the data. We advocated for measurement models with monotonic item ordering, such as the Rasch model, because they are more clearly grounded on principles of fundamental measurement. We also noted that if guessing was to be incorporated, then it would need to be modelled uniformly for all items, so that item curves did not overlap. Having disclosed our hand, a proponent of 2 and 3 PL-IRT models could challenge us and say, “*Right. We have a theory – we think that guessing is going to impact everyone, and we think that not all items will work the same way for all people. That is why we parameterised them. And doing that is not so different than saying*, ‘We think there will be differences between people depending on source, context, and dimension,’ *and modelling that.*”

Allowing the discrimination parameter to vary undercuts the notion of an invariant scale that takes on the same meaning for all participants, and puts us in a situation where each participant could potentially have their own scale with its own meaning that is not necessarily directly comparable to any other test taker. It is this situation that we are arguing against in this article. It is not clear why a test developer would intentionally design an item that was going to have, say, a 0.3 discrimination parameter and another item that was similarly theoretically designed to have a 0.7 discrimination parameter. Further, even if there was a substantive rationale, we suspect it would be almost impossible to achieve this in practice (it is hard enough to do this for difficulty parameters). In short, correcting for the “reality” of what each item’s discrimination turns out to be after the fact seems to be capitalising on luck rather than approaching the task informed by theory. One’s intention is critical. To make sense of our observations of the world, we develop models of relationships in our data. These models often begin by mapping the observed structure with the structure of potential analogues we already know (Dunbar and Blanchette, 2001). We might explore a number of models (i.e., analogues) before settling on a stable model that well-captures the theoretical underpinnings of the intended latent attribute. However, it would be unsatisfying if every time we considered that attribute, we needed to seek out a new model, which a data driven approach to measurement would seem to dictate. As a relevant side, such psychometric flexibility does little to address the current theory and replication crisis faced by some areas of psychological science (Proulx and Morey, 2021).

We presented two areas in our own work where method effects have been explicated as part of the experimental procedure. First, it is important for researchers to recognise that reactivity to observation is inherent in psychological measures as a starting point. Our research on reactivity to metacognitive prompts has demonstrated that the direction of such effects is not necessarily monotonic in nature. When this is the case, simple aggregation across observations will not result in appropriate solutions. It is therefore incumbent on the research discipline to expect exceptions, such as moderation and mediation effects that qualify summary accounts, and to not be satisfied with general effects. Research on context, source, and person-level effects indicates that such factors are important to the way we operationalise our latent attributes. The burgeoning research being conducted in both personality and cognitive areas aiming to more fully understand the sources and nature of within-person variability highlights the importance of being judicious in deciding the level of aggregation.

### A Final Comment on Method Effects

We accept that our definition of method effects is necessarily encompassing, rather than limiting. We do mean *any source of variance that is induced by the design or measurement* can act as a method effect, and in doing so consider other systematic factors/variables that moderate the assessment which are external or independent of the core observation. The first case study we present is a research design which includes two features, the presentation of a test item requiring (1) a response and (2) a confidence in correctness rating. For current purposes, the primary validity of measurement question (commonly framed as “construct validity”) concerns the test-item response. While the confidence rating is in principle independent of the item response, the reactivity research we cite demonstrates this is not necessarily the case. In practice, when asking participants to reflect on the accuracy of their item responses, we have introduced a method effect to the research design, which has a differential impact on individuals’ performances (Double and Birney, 2019b).

The second case study demonstrates the impact of situational contexts and observational sources in a research design investigating means and intra-person variability in personality ratings. The fact that the research design has substantive theoretical interest does not relinquish us from the responsibility to question our methods. Like the reactivity example, because the design (i.e., manipulations of context and source) introduces questions regarding validity of measurement (of personality in this case), we consider these factors as potential sources of method effects. Now one might wish to introduce some intentionality into the criteria for determining method effect status and argue that because we *intended* to manipulate context and source, these are theoretically substantive “design variables” rather than sources of method effects per se, and there is possibly some value in such a distinction. However, if these design variables result in either a change in the psychometric properties of the attribute assessed (e.g., additivity is diminished in one situation, as evidence by, for instance, poorer fit of data to the Rasch model) or more critically, a change in the actual attribute assessed (Birney and Sternberg, 2006), our methods have had an impact on assessment and this needs to be formally explicated *ex ante*. In sum, if a manipulation has an impact on the psychometric properties of measurement, then it is a method effect. We argue that substantive variables (like context and “focus of analysis”) can act as method effects and should be considered because they have the potential to impact the validity of measurement.

Increasing psychometric flexibility does not mean psychometric freedom without costs. In addressing Michell’s (1990) instrumental task, we should not lose sight of the scientific one, which is to well-understand and represent the quantifiable structure of our attributes in our measures. Investigations of method factors have great potential to allow us to more validly assess “old” constructs, but the approaches will also enable the identification and assessment of new ones. In short, method factors can be a threat or a windfall, but in either case, knowing how to identify, control and exploit them is key.

## Ethics Statement

The studies involving human participants were reviewed and approved by the Human Ethics Review Committee University of Sydney and University of New South Wales. The patients/participants provided their written informed consent to participate in this study.

## Author Contributions

DB conceived the general idea and wrote the first draft. DB, JB, NB, and SS contributed equally to explicating the theoretical underpinnings. All authors contributed to the article and approved the submitted version.

## Author Disclaimer

The views expressed herein are those of the authors and are not necessarily those of the Australian Research Council.

## Conflict of Interest

The authors declare that the research was conducted in the absence of any commercial or financial relationships that could be construed as a potential conflict of interest.

## Publisher’s Note

All claims expressed in this article are solely those of the authors and do not necessarily represent those of their affiliated organizations, or those of the publisher, the editors and the reviewers. Any product that may be evaluated in this article, or claim that may be made by its manufacturer, is not guaranteed or endorsed by the publisher.

## Footnotes

1 It could be argued that “Rasch” and “IRT” are redundant terms, especially for 1PL IRT models. However, we maintain the distinction here because it is substantive to our argument. Consistent with others (Andrich, 2003), use of a “1PL IRT” label gives the appearance that the choice to move to a 2- or 3-PL IRT model to improve fit of person responses to a model is routine, whereas it should not be. Further to this, Masters (1988) demonstrates how high (relative to low) item-discrimination can obscure the presence of distinct but positively correlated factors impacting on person-estimates. In some cases this leads to well-discriminating items that would often be considered ideal in a 2PL model, flagged as problematic in item-fit statistics in a Rasch model. The Rasch model would identify these as mis-fitting items to be investigated. 2+ PL models would give an apparent but ultimately unwarranted solution to the problem. Unwarranted because the 2+PL models would obscure the multi-dimensionality.

2 In the Rasch model, discrimination slopes of all items are constrained to equal 1, the slope of the natural logistic ogive, whereas the 1-PL IRT models fixes 1.7, which is the slope of the cumulative normal ogive.

3 There are two points here to make. First, it seems that when researchers model the guessing parameter, it tends to be in conjunction with the inclusion of discrimination as part of a 3PL-IRT model. In spite of this, we are aware of no reason why just “guessing” and item difficulty could be modelled without the problematic discrimination parameter. The second point is to recall Ben Wright’s counter-argument that items do not guess, people do, bringing into question whether it should be included at all (Wright, 1988).

4 Another description of the design of this research is as combined experimental-differential, which was referred to by Pedhazuer and Schmelkin (1991) as aptitude-treatment-interactions design, following on from Cronbach (1957).
